# A Digital Music-Based Mindfulness Intervention (“healing attempt”) for Race-Based Anxiety in Black Americans

**DOI:** 10.2196/51320

**Published:** 2023-10-12

**Authors:** Grant Jones, Franchesca Castro-Ramirez, Taylor McGuire, Maha Al-Suwaidi, Felipe Herrmann

**Affiliations:** 1 Department of Psychology Harvard University Cambridge, MA United States; 2 Department of Psychology University of California, Los Angeles Los Angeles, CA United States

**Keywords:** Black music, mindfulness, meditation, music, song, songs, psychotherapy, self-compassion, ethnic, cultural, culturally, single-case experiment, race, racial, anxiety, digital health intervention, Black, digital health, low income, Black community, racial disparity, mental health

## Abstract

This study replicates and extends findings that “healing attempt”—a brief digital music-based mindfulness intervention—represents a feasible and potentially effective intervention for race-based anxiety in the Black community.

## Introduction

Anxiety stemming from racism and discrimination disproportionately impacts Black Americans [1]. Decades of research indicate that mindfulness-based interventions can effectively alleviate anxiety [2]. However, three limitations often hinder the Black community from accessing these resources: a lack of cultural relevance, high costs, and excessive time commitments [3-5]. In response to these barriers, Author GJ created “healing attempt,” a brief digital music-based mindfulness intervention for race-based anxiety in the Black community. The intervention consists of originally composed and prerecorded songs, meditations, and poems. All components of the intervention are accompanied by background music rooted in Black music traditions (eg, gospel) to increase the cultural relevance of the intervention. Additionally, the intervention includes contributions from Lama Rod Owens, an acclaimed Buddhist teacher, and Terry Edmonds, former chief speechwriter for President Clinton. In light of the “replication crisis” in psychology [6], this study seeks to replicate and extend findings from our initial study in which we demonstrated that “healing attempt” may be a feasible and effective intervention for Black American race-based anxiety.

## Methods

### Overview

We recruited participants using Prolific—a web-based study platform—in January 2023. Participants were 8 middle-to-low income (<US $50,000/yr annual salary) Black American adults with elevated race-based anxiety. Study visits occurred on Zoom (Zoom Video Communications). We used a multiple-baseline design in which we assessed state anxiety and mindfulness/self-compassion every 2 minutes across two phases. In the initial phase (phase A), participants were randomized to one of four baseline conditions that ranged between 10 and 16 minutes (5-8 assessment periods). Participants were then instructed to go about their business as usual. In the second phase (phase B), we delivered the 25-minute intervention. The only instructions were to find a comfortable body position and attend to the intervention. After the intervention, we assessed the feasibility of the intervention using mixed methods.

The research team administered the questionnaires during the study. We used the State-Trait Anxiety Inventory–6 to assess state anxiety, and used a 5-item study-specific scale to measure state mindfulness/self-compassion; this scale includes items from the Toronto Mindfulness Scale and the State Self-Compassion Scale.

We analyzed all results in R (version 4.1.2; R Foundation for Statistical Computing) using the *Scan* package [7]. We used tau-U analyses to analyze our data; this analytical approach allows one to control for data trends when assessing intervention effects in multiple baseline studies, meaning that we could control for natural changes in anxiety or mindfulness/self-compassion during the study.

### Ethical Considerations

All study procedures were approved by the Harvard Institutional Review Board (protocol IRB21-0256). Participant data were deidentified. All participants provided informed consent before the study and were compensated with US $60.

## Results

Table 1 presents the results of our tau-U models assessing the impact of the intervention on state anxiety. All tau-U values were negative and significant (*P*<.001), including those that corrected for trends in the baseline and intervention phases, indicating that the intervention reduced state anxiety.

Table 2 presents the results of our tau-U models assessing the impact of the intervention on mindfulness/self-compassion. All tau-U values for these models were positive and significant (*P*<.05), suggesting that the intervention increased mindfulness/self-compassion.

Lastly, the feasibility scores for the intervention were high (ie, the average scores for all feasibility and acceptability measures were ≥86 out of 100). Further details on our results are provided in Multimedia Appendix 1.

**Table 1:** Overall tau-U models assessing the impact of the intervention on state anxiety.

Model		τ	SE	95% CI	*Z*	*P* value
**Overall tau-U (cases 1-4)**						
	A vs B	–0.91	0.14	–1.18 to –0.64	–6.57	<.001
	A vs B – trend A	–0.81	0.17	–1.14 to –0.49	–4.89	<.001
	A vs B + trend B	–0.73	0.10	–0.91 to –0.54	–7.62	<.001
	A vs B + trend B – trend A	–0.58	0.08	–0.74 to –0.42	–7.04	<.001
**Overall tau-U (cases 5-8)**						
	A vs B	–0.90	0.14	–1.17 to –0.63	–6.51	<.001
	A vs B – trend A	–0.79	0.17	–1.12 to –0.47	–4.79	<.001
	A vs B + trend B	–0.71	0.10	–0.90 to –0.52	–7.45	<.001
	A vs B + trend B – trend A	–0.56	0.08	–0.72 to –0.40	–6.77	<.001

**Table 2:** Overall tau-U models assessing the impact of the intervention on mindfulness/self-compassion.

Model		τ	SE	95% CI	*Z*	*P* value
**Overall tau-U (cases 1-4)**						
	A vs B	0.72	0.14	0.45-0.99	5.20	<.001
	A vs B – trend A	0.79	0.17	0.46-1.11	4.76	<.001
	A vs B + trend B	0.59	0.10	0.40-0.78	6.18	<.001
	A vs B + trend B – trend A	0.55	0.08	0.38-0.71	6.63	<.001
**Overall tau-U (cases 5-8)**						
	A vs B	0.47	0.14	0.20-0.74	3.43	<.001
	A vs B – trend A	0.39	0.17	0.06-0.71	2.33	.02
	A vs B + trend B	0.44	0.10	0.26-0.63	4.64	<.001
	A vs B + trend B – trend A	0.34	0.08	0.17-0.50	4.08	<.001

## Discussion

We replicated and extended our initial findings and showed that “healing attempt” can improve anxiety and mindfulness/self-compassion with high feasibility/acceptability [8]. Congruent with the existing literature, the observed improvements in mindfulness/self-compassion may have driven the reductions in anxiety [2,9]. Similarly, the culturally adapted nature of the intervention also may have contributed to symptom reduction and the high feasibility scores in this experiment [10].

Some limitations should be acknowledged. First, the small sample size and within-subject design limit the ability to make strong causal claims from the results. Second, our prescreening criteria requiring participants to have familiarity with meditation limits generalizability. Third, the mindfulness/self-compassion measure used in the study was not a fully validated scale. Fourth, in the qualitative feedback, one participant suggested eliminating the frequent pausing of the music that accompanied the study design.

We can address the aforementioned limitations in a future between-subjects randomized feasibility trial that tests “healing attempt” as a single-session intervention. Additionally, future studies can investigate novel low-cost avenues for disseminating the intervention, such as music-streaming platforms. Overall, this study represents a promising next step in providing a culturally relevant, highly scalable mindfulness resource for the Black community.

## Appendix

Multimedia Appendix 1 Supplementary materials.

## Data Availability

A link to the data and code for this project is available in Multimedia Appendix 1. No works have been published or submitted that stem from this data set.
