# Supplementary material for: A Digital Music-Based Mindfulness Intervention (“healing attempt”) for Race-Based Anxiety in Black Americans
Source: J Med Internet Res. 2023 Oct 12;25:e51320. doi: 10.2196/51320 (PMC10603556; doi:10.2196/51320)
Supplement: Multimedia Appendix 1 [file jmir_v25i1e51320_app1.docx]

The data and code for this project are available at the following web address: <https://osf.io/6e3qv/?view_only=067e0c36b09844248309ff684edae56d>

#### **Supplementary Material 1**

State Self-Compassion Scale Questions [Scale 1-5; 1- not at all true for me, 5- very true for me]:

-I’m giving myself the caring and tenderness I need.

-I feel intolerant and impatient toward myself. (reverse coded)

-I’m keeping things in perspective.

Toronto Mindfulness Scale Questions [Scale 0-4: 0- not at all, 4 - very much so]:

-I was curious about what I might learn about myself by taking notice of how I react to certain thoughts, feelings or sensations

-I was more invested in just watching my experiences as they arose, than in figuring out what they could mean.

**Supplementary Material 2**

Feasibility and Acceptability. Feasibility and acceptability were primarily assessed with seven study-specific items. Participants were asked the following:

1. How likely would you be to recommend this intervention to someone you care about from the Black community that is dealing with acute stress/anxiety? (Variable: “Likely to recommend”) (0: Not at all likely; 50: moderately likely; 100: Definitely likely)

2. To what extent was the intervention harmful or helpful for your stress/anxiety? (“Helpful for Anxiety”) (0: harmful; 50: no impact; 100: incredibly helpful)

3. To what extent did you find it difficult or easy to engage with the intervention? How practical and logistically easeful did it feel? (“Easy to Engage With”) (0: extremely difficult; 50: neutral; 100: as easy as I could imagine)

4. To what extent do you feel like the study changed your ability to be present with your thoughts and emotions? (“Improved Ability to be Present”) (0: much less able to be present; 50: no change; 100: much more able to be present)

5. To what extent do you think the study changed the way you feel about yourself? (“Improved Relationship with Self”) (0: much worse about myself; 50: no change; 100: much better about relationship to self)

6. To what extent do you feel that this intervention was made for people like you? (“Made for You”) (0: not at all; 50: somewhat; 100: exactly for me)

7. How helpful/harmful do you think it would be to experience and work with this intervention over a longer period of time, (i.e. daily for one to two weeks) (“Longer Engagement Helpful”) (0: very harmful; 50: no difference; 100: very helpful).

Additionally, we gathered additional qualitative and quantitative feedback on the intervention feasibility with the following questions as well:

1. If you believe a music-based mindfulness intervention like this one could be helpful for some people, how many days do you think one would need to engage with such an intervention to see lasting benefits for stress/anxiety? (“# of days for lasting benefit)
2. Is there anything you particularly liked about the study and/or intervention? Please feel free to let me know in a few simple words/sentences. (“Likes”)
3. Is there anything you particularly did not like about the study and/or intervention? Please feel free to let me know in a few simple words/sentences. (“Dislikes”)

#### **Supplementary Material 3**

#### Demographics of participants

| Participant | Gender | Age | Income | Education | Meditation Familiarity | Music for Support | Trait Anxiety (STAIT5) | Racism as a cause of anxiety |
| --- | --- | --- | --- | --- | --- | --- | --- | --- |
| Case 1 | Female | 30 | <$15,000 | Some college (left early) | moderately so (“I meditate on occasion”) | often | 12 | 60 |
| Case 2 | Male | 29 | $15,000-$24,999 | High school graduate | moderately so (“I meditate on occasion”) | all the time | 15 | 90 |
| Case 3 | Non-Binary | 34 | $15,000-$24,999 | Bachelor’s degree | somewhat (“I’ve meditated once or twice”) | often | 18 | 100 |
| Case 4 | Female | 28 | $35,000-$49,999 | Some college (left early) | somewhat (“I’ve meditated once or twice”) | all the time | 20 | 70 |
| Case 5 | Female | 38 | $35,000-$49,999 | Some college (left early) | extremely (“Meditation is one of the most important aspects of my life”) | often | 12 | 70 |
| Case 6 | Female | 42 | $15,000-$24,999 | Bachelor’s degree | moderately so (“I meditate on occasion”) | all the time | 20 | 60 |
| Case 7 | Male | 33 | $35,000-$49,999 | Associate’s degree | moderately so (“I meditate on occasion”) | often | 18 | 70 |
| Case 8 | Female | 35 | $15,000-$24,999 | Some college (left early) | very much so (“I have a regular meditation practice”) | often | 12 | 70 |

#### **Supplementary Material 4**

Anxiety scores (State-Trait Anxiety Inventory-6 [STAI-6]) for each of the 8 participants over the course of the study


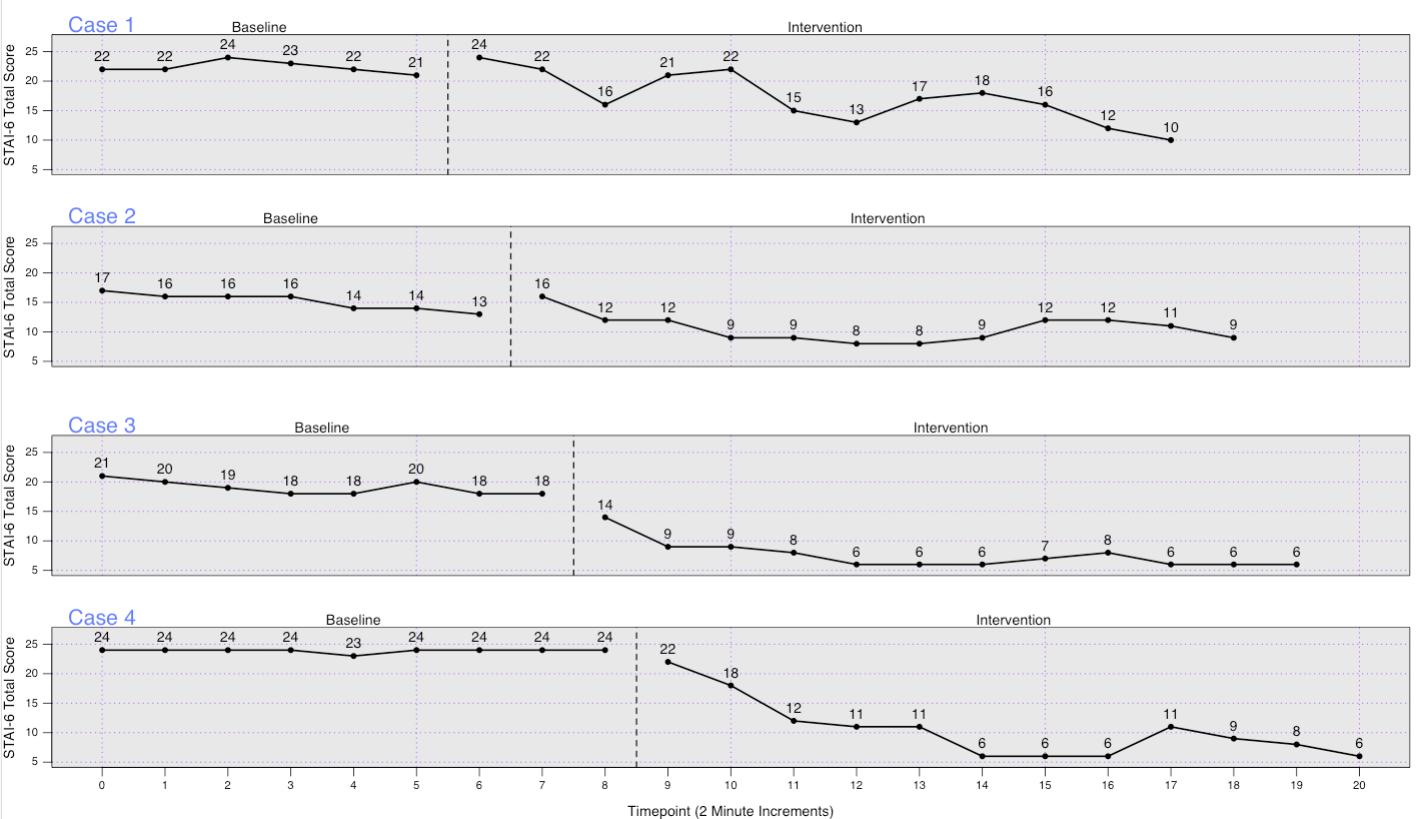


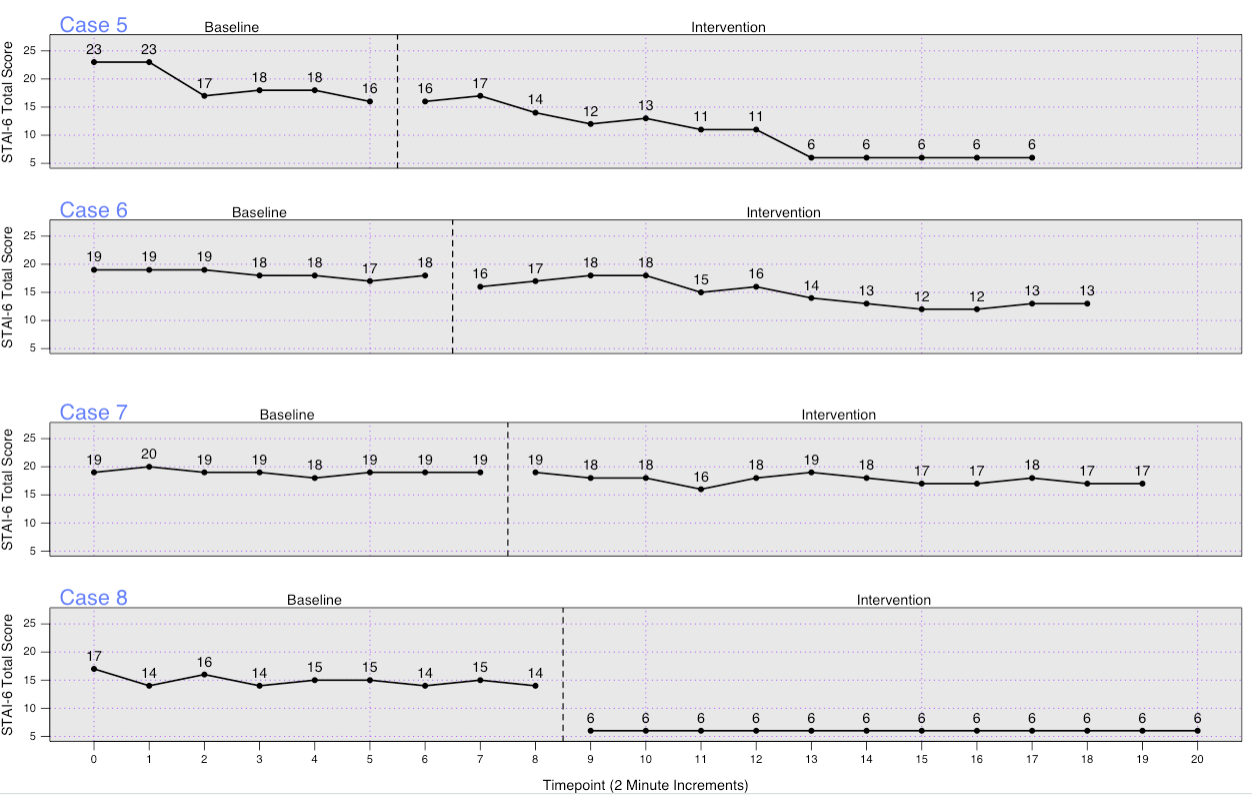


#### **Supplementary Material 5**

Mindfulness/self-compassion scores for each of the 8 participants over the course of the study.


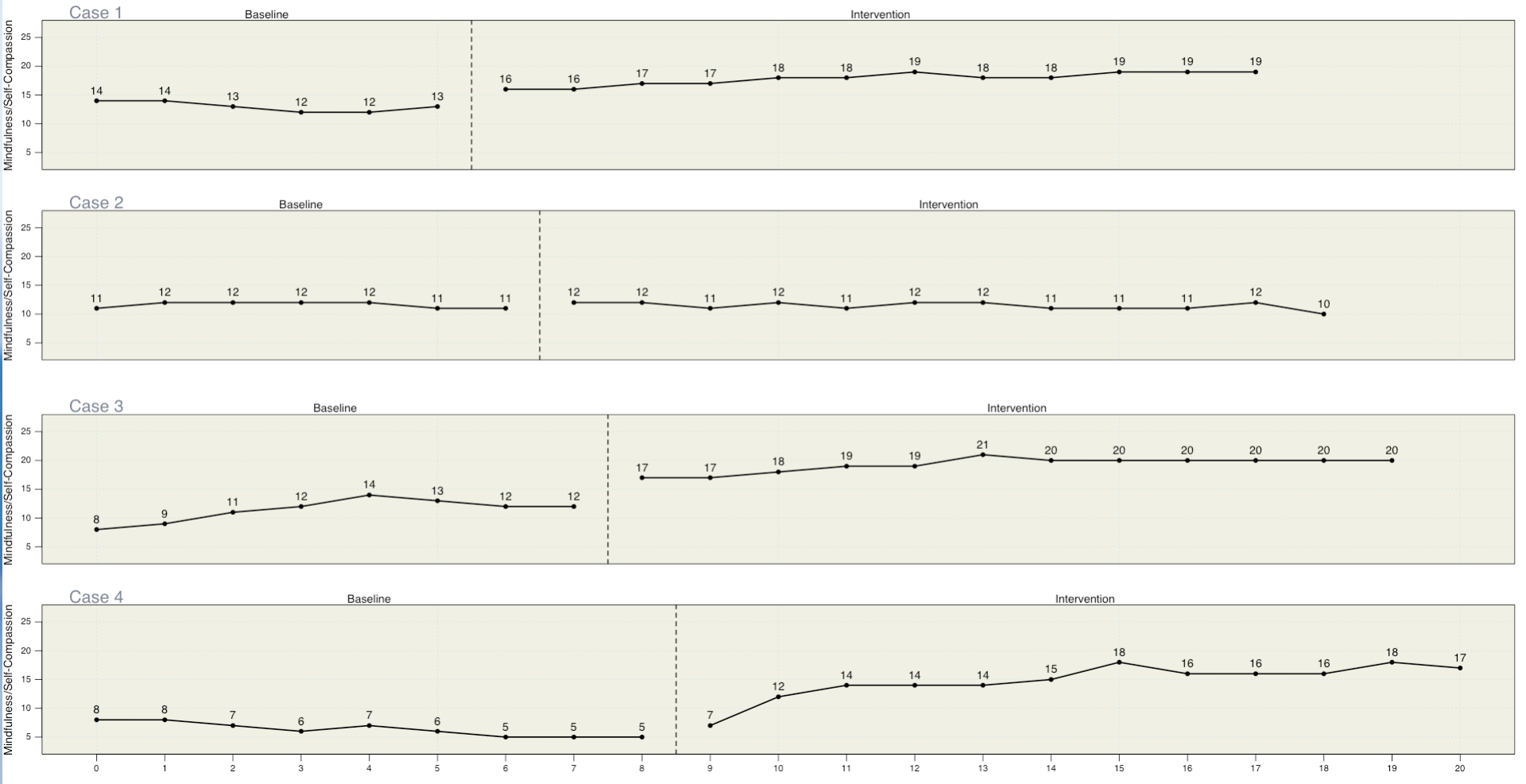


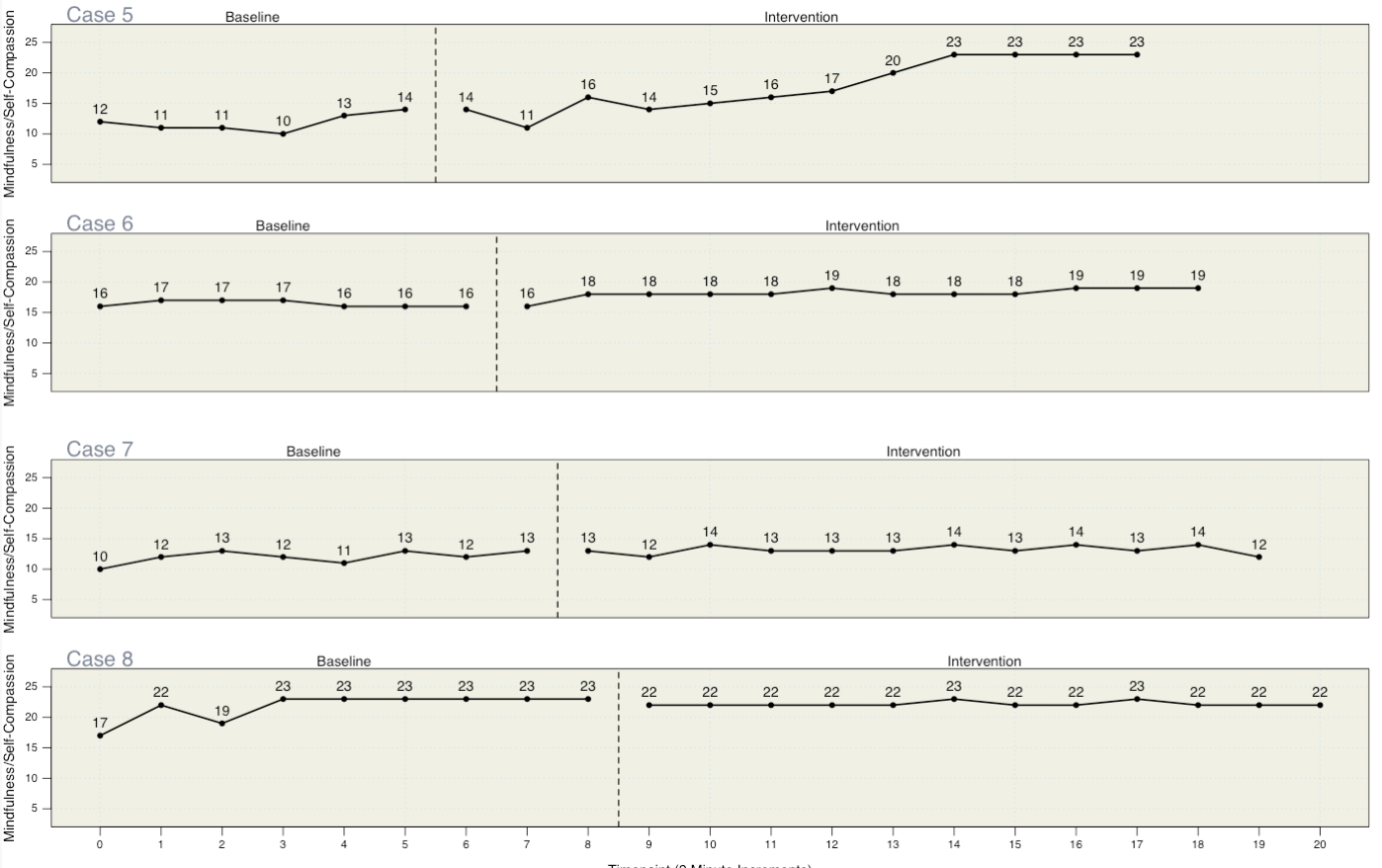


#### **Supplementary Material 6**

*Table 7.* Feasibility and acceptability assessments

| Participant | Likely to Recommend | Helpful for Anxiety | Easy to Engage With | Improved Ability to be Present with Emotions | Improved Relationship with Self | Made for You | Longer Engagement Helpful |
| --- | --- | --- | --- | --- | --- | --- | --- |
| Case 1 | 100 | 100 | 50 | 100 | 100 | 100 | 100 |
| Case 2 | 72 | 65 | 85 | 55 | 55 | 75 | 75 |
| Case 3 | 100 | 85 | 50 | 80 | 100 | 99 | 100 |
| Case 4 | 100 | 100 | 100 | 100 | 100 | 100 | 100 |
| Case 5 | 100 | 100 | 100 | 100 | 100 | 100 | 100 |
| Case 6 | 100 | 100 | 100 | 100 | 100 | 100 | 100 |
| Case 7 | 60 | 60 | 100 | 60 | 70 | 90 | 80 |
| Case 8 | 100 | 98 | 100 | 100 | 90 | 100 | 100 |
| Mean | 92 | 88 | 86 | 87 | 89 | 96 | 94 |

#### **Supplementary Material 7**

*Table 8.* # of days needed for lasting benefit, likes, and dislikes/suggestions for improvement

| Participants | # of Days for Lasting Benefit | Likes | Dislikes/ Suggestions for Improvement |
| --- | --- | --- | --- |
| Case 1 | 7 | Music; singing | None |
| Case 2 | 14 | Music; the simplicity of the intervention | Audio glitches |
| Case 3 | 14 | Music | None |
| Case 4 | 30 | Singing | None |
| Case 5 | 15 | Music | None |
| Case 6 | 52 | Black facilitators | None |
| Case 7 | 30 | Music | Pausing the audio |
| Case 8 | 30 | Music | None |

#### **Supplementary Material 8**

Power analysis for 5 baseline periods and 12 intervention periods for 3 participants to detect a moderate intervention effect (d = 0.5) using overall Tau U analyses. Conducted using 500 monte-carlo simulations.

| Method | Power | Alpha Error | Alpha:Beta | Correct | p-value |
| --- | --- | --- | --- | --- | --- |
| Overall Tau-U Analyses | 84% | 4% | 1:4.1 | 89.9 | 0 |
